# Association of Pathways to Success Launch With Quality inBeneficiaries With Traditional Medicare

**DOI:** 10.1111/1475-6773.70024

**Published:** 2025-07-31

**Authors:** Meiling Ying, Addison Shay, Richard A. Hirth, John M. Hollingsworth, Vahakn B. Shahinian, Brent K. Hollenbeck

**Affiliations:** ^1^ Department of Foundations of Medicine NYU Langone Health Mineola New York USA; ^2^ Dow Division of Health Services Research, Department of Urology University of Michigan Ann Arbor Michigan USA; ^3^ Department of Health Management and Policy University of Michigan Ann Arbor Michigan USA; ^4^ Department of Urology University of Florida Gainesville Florida USA

**Keywords:** accountable care organization, pathways to success, quality of care, two‐sided risk model

## Abstract

**Objective:**

To evaluate the association between implementation of “Pathways to Success” and quality among beneficiaries cared for in Shared Savings Program accountable care organizations (ACOs).

**Study Setting and Design:**

Medicare initiated “Pathways to Success” in 2019 that required upside‐risk only ACOs in Shared Savings Program to transition to a two‐sided risk model and prior two‐sided ACOs to assume even greater financial responsibility. We examined the association between Pathways and ACO‐targeted (hospitalizations for congestive heart failure [CHF] and all‐cause 30‐day readmissions) and nontargeted (all‐cause emergency department visits without hospitalization for CHF and hospital observation stays) quality measures, using a difference‐in‐differences framework.

**Data Sources and Analytic Sample:**

Data were extracted from a 20% sample of national Medicare data from 2018 to 2020. This study included 810,070 beneficiary‐quarters in 514 ACOs, and 813,855 beneficiary‐quarters never attributed to an ACO (i.e., controls).

**Principal Findings:**

Implementation of Pathways was not associated with significant relative changes in the quarterly number of CHF admissions (decreasing from 97.98 to 82.04 per 1000 beneficiaries in ACOs; differential change = 3.51 quarterly CHF admissions per 1000 beneficiaries, 95% CI, −4.82 to 11.85) or the quarterly number of emergency department visits for CHF (decreasing from 110.90 to 97.50 per 1000 beneficiaries in ACOs; differential change = 6.47 quarterly CHF emergency department visits per 1000 beneficiaries, 95% CI, −3.71 to 16.64). However, quarterly rates of 30‐day all‐cause readmissions increased slightly by 0.61% points (95% CI, 0.23 to 0.98; unadjusted readmissions increased from 14.49% to 14.81% in ACOs) after Pathways implementation. Observation stays remained unchanged (differential change = −0.16% points, 95% CI, −0.33 to 0.02; unadjusted observation stays increased from 3.64% to 3.94% in ACOs) after the launch of Pathways.

**Conclusions:**

Medicare's Pathways to Success, which introduced two‐sided risk, was not associated with improvement in select quality measures.

**Summary**

- What is known on this topic
  ○ Before 2019, the Shared Savings Program offered four different tracks with varying levels of financial risk and reward, with 82% of ACOs participating in upside‐only models.
  ○ To encourage broader participation in risk‐bearing models, Medicare introduced the “Pathways to Success” program in December 2018, implementing it in July 2019.
  ○ While the Shared Savings Program led to improvements in quality, the impact of Pathways on quality outcomes, considered a measure of the payment model's success, remains unclear.
- What this study adds
  ○ Pathways to Success was not associated with significant changes in care access outcomes, as measured by quarterly hospitalizations for CHF (an ACO targeted measure) and quarterly emergency department visits for CHF (an ACO nontargeted measure).
  ○ Pathways did not significantly affect observation stays for ACO beneficiaries; however, 30‐day all‐cause readmissions—a targeted measure of the Shared Savings Program—increased after the Pathways to Success launch.
  ○ Pathways to Success, which introduced two‐sided risk, was not associated with improvement in select quality measures.

## 1 Introduction

The Shared Savings Program consists of 456 accountable care organizations (ACOs) serving approximately 10.9 million beneficiaries with Traditional Medicare in 2023 [1]. Prior to 2019, the Shared Savings Program provided four separate tracks with varying levels of financial risk and reward. As of 2018, 82% of ACOs participated in “Track 1” [1], a model that allowed participants to share in savings with Medicare if spending per beneficiary was below a specified benchmark and quality goals were achieved. As an upside‐only model, Track 1 ACOs faced no penalties for failing to achieve benchmarks. This model led to improvements in quality but increased overall Medicare spending due to distribution of the savings [2, 3, 4, 5, 6, 7, 8]. To broaden participation in risk bearing models, Medicare announced the “Pathways to Success” program in December 2018 and implemented Pathways in July 2019 [9]. Pathways consolidated the Shared Savings Program from the previous four tracks into two, requiring Track 1 ACOs to transition to a two‐sided risk model—one which has both upside and downside risk—in as little as 1 year [9]. Furthermore, preexisting two‐sided ACOs were required to enroll in Enhanced tracks, which offered higher percentages of shared savings but also significantly increased the risk of financial losses [9].

The intent of Pathways to Success was to globally rachet up the opportunity for reward and the risk of loss, thereby increasing the “skin in the game” of Shared Savings Program participants. However, the relationships between Pathways and quality, viewed as a success of the payment model, are uncertain. A survey‐based study showed that prior to Pathways implementation, ACOs performed well on measured domains of quality (e.g., screening for fall risk and depression, pneumonia vaccinations between 2013 and 2016) [10], due to substantial investment in data systems, analytical support, and care coordination capabilities [11]. Under Pathways, increased accountability and the possibility of penalties may encourage ACOs to unilaterally focus resources on measures targeted by the program [12, 13], ignoring other clinically important areas. Alternatively, prompted by the looming possibility of penalties, incremental investment in system‐level capabilities to monitor quality and/or coordinate care delivery may enhance quality, both within and outside the purview of the Shared Savings Program. On the other hand, Pathways could prompt high‐performing ACOs to withdraw from the program, potentially failing to improve or even worsen overall clinical quality [14].

For these reasons, we performed a retrospective cohort study using national Medicare data from 2018 to 2020 to evaluate the overall associations of Pathways to Success implementation with quality in domains targeted and untargeted by the Shared Savings Program.

## 2 Methods

### 2.1 Study Design and Overview

We used a 20% sample of national Medicare data from January 1, 2018, through March 31, 2020 (i.e., pre‐pandemic), to measure adherence to quality measures before and after Pathways implementation on July 1, 2019. Using a difference‐in‐difference model, we contrasted changes in each quality measure among beneficiaries served by ACOs (i.e., the ACO group) and those served by nonparticipating physicians (i.e., the control group). To assess for heterogeneity in effects across quality domains within and outside the scope of the Shared Savings Program, we assessed aspects of quality that were targeted and untargeted by the program.

The study population both within and outside of ACOs consisted of beneficiaries having at least one office visit in a study year with a primary care physician. Per the SSP policy [15] and based on prior research [5, 6, 7], specialty codes within the data were used to identify primary care physicians and included: general practice (01), family practice (08), internal medicine (11), pediatric medicine (37), geriatric medicine (38), nurse practitioner (50), clinical nurse specialist (89), or physician assistant (97). We then included those continuously enrolled in Medicare Parts A and B in each quarter (or while alive in the case of decedents) and in the preceding 12 months (to assess preexisting clinical conditions). We also limited the study cohort to those residing in hospital referral regions [16] to improve comparability between ACO beneficiaries and controls.

We next attributed eligible beneficiaries to their primary care physician using the plurality of allowed charges for office visits, determined annually. From the ACO Provider‐level Research Identifiable File, primary care physicians were then assigned to ACOs using taxpayer identification numbers (for acute‐care hospitals) and Centers for Medicare and Medicaid Services Certification Numbers (for safety‐net providers [including rural health centers, critical access hospitals, and federally qualified health centers]) [17]. Beneficiaries assigned to an ACO between January 1, 2018, and March 31, 2020, served as the group exposed to the policy. The remaining beneficiaries never aligned to an ACO between 2018 and 2020 served as controls.

### 2.2 Quality Measures

There were four quality measures of interest in our study. We assessed two pairs of quality measures capturing different domains—care access and care coordination. For each pair of measures, one measure was targeted by the Shared Savings Program and the other was not. The first quality domain assessed was related to access to timely ambulatory care (i.e., access). The measure targeted by the Shared Savings Program was the quarterly rate of hospital admissions in patients with a diagnosis for congestive heart failure (CHF), the most common ambulatory care–sensitive condition and one for which appropriate ambulatory care could reduce the need for admission [5, 18, 19]. For this measure, the numerator comprised the number of admissions with a principal diagnosis of CHF, measured quarterly, with the denominator determined by the number of beneficiaries aged 66 and older with CHF. Admissions transferred to other healthcare institutions were censored from the numerator [20]. We hypothesize that Pathways would reduce admissions relative to controls. To evaluate the possibility that Pathways might encourage less optimal alternatives to hospitalization, we measured quarterly rates of emergency department visits for CHF without hospitalization among beneficiaries aged over 65 with CHF. As this measure was not included in ACO contracts, we expected that Pathways implementation would increase visits for those managed by ACOs relative to controls.

For the care coordination domain, the targeted measure consisted of 30‐day all‐cause, unplanned readmissions (assessed as yes/no) for previously hospitalized patients [3, 21]. This outcome is a key focus of the Shared Savings Program as well as other major ongoing (e.g., the Bundled Payments for Care Improvement Advanced Model [22]) and forthcoming (e.g., the States Advancing All‐Payer Health Equity Approaches and Development Model [23]) alternative payment models that incorporate downside risk. Therefore, understanding the impact of introducing downside financial risk into a value‐based payment model may help inform the design of both current and future models. The denominator included all eligible beneficiaries aged 66 years and older hospitalized and discharged alive from non‐federal, short‐stay, acute‐care or critical access hospitals. The numerator was determined by the number of beneficiaries in the previously defined denominator that had an unplanned readmission for any cause within 30 days. For multiple unplanned readmissions during the 30‐day period, each beneficiary was counted only once. Readmissions to the same hospitals on the same day for the same principal diagnosis were excluded. Beneficiaries hospitalized for medical treatment of cancer, primary psychiatric diagnoses, or rehabilitation, fitting of prostheses and adjustment devices, and discharged to other acute care hospitals or against medical advice were also excluded [24]. We hypothesized that Pathways would reduce readmissions in ACOs relative to controls. As reductions in readmissions might be achieved by observing a patient without readmission, circumventing the policy, we examined changes in rates of observation stays, a care coordination measure not targeted by the Shared Savings Program. We used the same denominator as above, but limited the numerator to the number of patients requiring an observation stay, defined by the revenue center code 0732 in the outpatient file, within 30 days of their initial hospital discharge [4, 25, 26]. Only one observation stay was included per beneficiary [4]. We expect that Pathways would lead to greater use of observation stays relative to controls.

### 2.3 Statistical Analysis

We compared pre‐Pathways beneficiary characteristics between ACOs and controls using two tailed $\chi^{2}$ tests for categorical variables and t tests for continuous variables. A difference‐in‐differences design was used to investigate the association between Pathways implementation and each quality measure for beneficiaries in ACOs relative to controls (eA. Model Specification in Supplement). For the access to care measures (i.e., count outcomes), assessed as rates per 1000 eligible Medicare beneficiaries, we fitted patient‐level generalized linear models with a Gaussian family function and an identity link function following prior research [5]. For the care coordination measures (i.e., binary outcomes), assessed as percentages, we estimated patient‐level generalized linear models with a binomial family function and a logit link function [5]. All models included an indicator for those in and outside of ACOs, a binary indicator for the time period—“pre” (i.e., January 1, 2018, to September 30, 2018) versus “post” (i.e., July 1, 2019, to March 31, 2020)—and an interaction term between ACO and time variables. The “pre” period was selected to avoid potential preemptive action taken before the official announcement of Pathways to Success on December 21, 2018 (i.e., October 1, 2018, through June 30, 2019, served as a washout period). The “post” period was chosen to capture Pathways to Success's effective date (i.e., July 1, 2019) and avoid conflating influences from the pandemic [9, 27]. Models were adjusted for beneficiary characteristics, including age, sex, race or ethnic group (categorized as Black, non‐Hispanic white, and others [including American Indian/Alaska Native, Asian/Pacific Islander, Hispanic, and other individuals], determined by the Research Triangle Institute Race code in the Medicare enrollment file) [28], comorbidity, dual eligibility [29, 30], and indicators as to whether end‐stage renal disease or disability were the origin of Medicare eligibility [5, 7]. Comorbidity was estimated using Hierarchical Condition Category (HCC) risk scores [5, 7, 31]. Finally, models were additionally adjusted for Health Service Area‐level characteristics from the American Community Survey DATA [32], including the percentages of residents falling below the federal poverty level, holding high school diplomas, and those with college degrees to capture Health Service Area‐level sociodemographic characteristics [5, 7]. Following prior research, models included fixed effects for each ACO to account for pre‐Pathways differences between the ACO and control groups and for changes in the distribution of ACO‐aligned beneficiaries across ACOs. Fixed effects for each hospital referral region (HRR) were used in each quarter to adjust for HRR‐specific changes in clinical care for controls and to compare differences between ACO‐attributed beneficiaries and beneficiaries served by the control group living in the same area [5, 7]. Due to the voluntary nature of the Shared Savings Program, models were further adjusted for the probability of participating in an ACO using inverse probability weights calculated by logistic regressions (eA. Model Specification in Supplement) to balance observed patient characteristics between ACO and control groups during the pre‐intervention period [5, 7]. The quantity of interest was the regression coefficient for the interaction between the indicator for group (ACO vs. control) and time (pre vs. post). This interaction estimated the average differential change in the quality in beneficiaries in ACOs relative to controls.

We tested the parallel trends assumption of our difference‐in‐differences approach by comparing trends in each quality measure between ACOs and controls in the pre‐implementation period statistically and graphically. All the outcomes met the modeling requirements (eTable 1 and eFigure 1 in Supplement).

The sensitivity of the significant findings to several different specifications was examined, including an alternative definition of the preintervention (i.e., July 1, 2017–March 31, and July 1, 2017–September 30,2018) and postintervention (i.e., January 1, 2019–March 31, 2020) periods, repeating the main analysis with an alternative estimation method (i.e., generalized linear models with a Gaussian family function and an identify link function), by incorporating beneficiaries with unknown race/ethnicity, restricting the study period to data through March 12, 2020—one day before the White House officially declared COVID‐19 a global pandemic, and modifying the outcome definitions (i.e., converting binary outcomes to count outcomes and including observation stays in the denominator for readmission outcomes [33]).

There were no missing data. All analyses were performed using STATA version 15.1 and reported the robust estimator of standard errors to account for the clustering of beneficiaries within ACOs (for the ACO group) or hospital referral regions (for the control group) [5, 34]. A two‐tailed test with a *P*‐value of < 0.05 was considered statistically significant. We reported the marginal effects of estimates for the ease of interpretation of the multivariable regression models. The study was determined not to be human participants research by the University Institutional Review Board, which waived the requirement of informed consent. This observational study followed the Strengthening the Reporting of Observational Studies in Epidemiology (STROBE) reporting guidelines.

## 3 Results

Our cohort included 810,070 beneficiary‐quarters in 514 ACOs, and 813,855 beneficiary‐quarters in the control group. Table 1 illustrates the differences in baseline characteristics between the ACO and controls before and after inverse probability of treatment weighting. We observed significant but small differences in beneficiaries' demographics and clinical characteristics, and Health Service Area‐level sociodemographic attributes between ACOs and controls (i.e., the “Actual” column). These differences were further minimized after the inverse probability of treatment weighting. For instance, the percentage of beneficiaries with full Medicaid benefits was substantially lower in ACOs (7.1%) than in controls (12.9%). After adjusting for inverse probability of treatment weighting, the gap narrowed to 8.9% in ACOs versus 11.0% in controls. The corresponding standardized mean differences decreased from −0.19 to −0.07.

**TABLE 1:** Patient and market characteristics among beneficiaries in ACOs and controls in the “pre” Pathways period (January 1, 2018, to September 30, 2018) before and after inverse probability of treatment weighting.

Characteristic	Actual (*N* = 262,401 unique beneficiaries)			After inverse probability of treatment weighting (*N* = 262,401 unique beneficiaries)		
	ACO (*N* = 123,437)	Controls (*N* = 138,964)	SMD	ACO (*N* = 123,437)	Controls (*N* = 138,964)	SMD
Age, mean (SD)	79 (8.1)	79 (8.4)	0.00	79 (8.1)	79 (8.4)	0.01
Female, (%)	70,217 (56.9%)	78,814 (56.7%)	0.00	70,202 (56.9%)	78,902 (56.8%)	0.00
Race/ethnicity, (%)						
Black	9.088 (7.4%)	11,650 (8.4%)	−0.04	9750 (7.9%)	10,944 (7.9%)	0.00
Non‐Hispanic white	106,942 (86.6%)	112,949 (81.3%)	0.15	104,173 (84.4%)	16,473 (83.8%)	0.02
Other^a^	7.407 (6.0%)	14,365 (10.3%)	−0.16	9514 (7.7%)	11,547 (8.3%)	−0.02
Medicare‐Medicaid dual eligibility, (%)						
Medicare‐only	105,332 (85.3%)	106,260 (76.5%)	0.23	100,472 (81.4%)	111,421 (80.2%)	0.03
Full	8804 (7.1%)	17,912 (12.9%)	−0.19	10,951 (8.9%)	15,295 (11.0%)	−0.07
Partial	9301 (7.5%)	14,792 (10.6%)	−0.11	12,014 (9.7%)	12,248 (8.8%)	0.03
End‐stage renal disease, (%)	3101 (2.51%)	3944 (2.84%)	−0.02	3155 (2.56%)	3689 (2.65%)	−0.01
Disability as reason for eligibility, (%)	15,809 (12.8%)	20,388 (14.7%)	−0.05	16,779 (13.6%)	19,159 (13.8%)	−0.01
HCC risk score, (%)						
0	15,953 (12.9%)	19,102 (13.8%)	−0.02	16,408 (13.3%)	18,603 (13.4%)	0.00
1	20,179 (16.4%)	22,444 (16.2%)	0.01	19,985 (16.2%)	22,599 (16.3%)	0.00
2	19,159 (15.5%)	20,989 (15.1%)	0.01	18,780 (15.2%)	21,279 (15.3%)	0.00
≥ 3	68,146 (55.2%)	76,429 (55.0%)	0.00	68,264 (55.3%)	76,483 (55.0%)	0.01
% below federal poverty level, mean (SD)	15.0% (5.5%)	16.0% (6.0%)	−0.18	15.5% (5.7%)	15.7% (5.8%)	−0.04
% high school diploma, mean (SD)	31.6% (5.9%)	31.6% (6.5%)	−0.01	31.6% (6.2%)	31.7% (6.4%)	−0.03
% college degree, mean (SD)	9.9% (5.5%)	9.4% (5.9%)	0.10	9.6% (5.7%)	9.4% (5.7%)	0.04

Abbreviations: ACO, Accountable Care Organization; SD, Standard Deviation; SMD, Standardized Mean Difference.

^a^ Other race/ethnicity groups include American Indian/Alaska Native, Asian/Pacific Islander, Hispanic, and other individuals.

In Table 2, the unadjusted quarterly CHF admissions per 1000 beneficiaries in ACOs decreased from 97.98 pre‐Pathways to 82.04 post‐Pathways, resulting in a reduction of 15.94 quarterly CHF admissions per 1000 beneficiaries. In controls, the unadjusted quarterly CHF hospitalizations per 1000 declined from 90.07 pre‐Pathways to 71.19 post‐Pathways, leading to a decrease of 18.88 quarterly CHF admissions per 1000 beneficiaries. The risk‐adjusted differences‐in‐differences analysis showed that the relative change in ACOs did not significantly differ from those in controls (differential change = 3.51 quarterly CHF admissions per 1000 beneficiaries, 95% CI, −4.82, 11.85).

**TABLE 2:** Relative changes in targeted and untargeted quality measures associated with Pathways to Success Implementation on July 1, 2019.

Quality measures	ACO			Controls			Difference‐in‐differences	
	Pre*	Post*	Unadjusted difference (*post—pre*)*	Pre*	Post*	Unadjusted difference (*post—pre*)*	Adjusted differential change (95% CI)*	*P*‐value
*Access to care*								
Admissions for CHF (targeted)	97.98	82.04	−15.94	90.07	71.19	−18.88	3.51 (−4.82, 11.85)	0.41
Emergency department visits for CHF (untargeted)	110.90	97.50	−13.40	105.68	88.15	−17.53	6.47 (−3.71, 16.64)	0.21

	Pre %	Post %	Unadjusted %difference (*post—pre*)	Pre %	Post %	Unadjusted % difference (*post—pre*)	Adjusted differential change (95% CI)^§^	*P*‐value
*Care Coordination*								
30‐Day all cause readmissions (targeted)	14.49	14.81	0.32	15.78	15.41	−0.37	0.61 (0.23, 0.98)	0.002
Observation stays (untargeted)	3.64	3.94	0.30	3.26	3.56	0.30	−0.16 (−0.33, 0.02)	0.08

Abbreviations: ACO, Accountable Care Organization; CHF, Congestive Heart Failure; CI, Confidence Interval.

* Expressed as rates per 1000 CHF beneficiaries.

^**§**^ Expressed as percentage points.

A similar pattern was detected in the quarterly emergency department visits for CHF. Unadjusted quarterly emergency department visits for CHF decreased by 13.40 per 1000 beneficiaries in ACOs (from 110.90 pre‐Pathways to 97.50 post‐Pathways) and by 17.53 per 1000 beneficiaries in controls (from 105.88 pre‐Pathways to 88.15 post‐Pathways). After the risk adjustment, the relative decline in quarterly emergency department visits was similar in ACOs and controls (differential change = 6.47 quarterly emergency department visits, 95% CI, −3.71, 16.64).

Before Pathways, the unadjusted quarterly rate of 30‐day all‐cause readmissions was 14.49% in ACOs and 15.78% in controls. After Pathways, this rate increased to 14.81% in ACOs but decreased to 15.41% in controls. This resulted in a modest relative increase in the quarterly 30‐day all‐cause readmissions following Pathways implementation (differential change = 0.61% points, 95% CI 0.23, 0.98). However, the relative change in use of observation stays after discharge was similar in ACOs and controls after Pathways (differential change = −0.16 percentage points, 95% CI −0.33, 0.02). Sensitivity analyses of the significant finding (i.e., relative increase in 30‐day all‐cause unplanned readmissions among ACOs) confirmed that it was robust to several different specifications (Table 3).

**TABLE 3:** Sensitivity analyses for quarterly 30‐day all cause unplanned readmissions.

Model identification strategy	Effect size in % (95% CI)
	Quarterly 30‐day readmissions
Main model specification (*n* = 1,510,352 beneficiary‐quarters)	0.61 (0.23, 0.98)
Alternative pre‐Pathways periods	
July 1, 2017 – March 31, 2018 (*n* = 1,476,945 beneficiary‐quarters)	1.19 (0.76, 1.62)
July 1, 2017 – September 30, 2018 (*n* = 1,971,716 beneficiary‐quarters)	0.90 (0.53, 1.26)
Alternative post‐Pathways period:	
January 1, 2019 – March 2020 (*n* = 2,236,590 beneficiary‐quarters)	0.61 (0.25, 0.96)
Alternative estimation approach: generalized linear models with a Gaussian family function and an identity link function (*n* = 1,510,352 beneficiary‐quarters)	0.64 (0.26, 1.01)
Including 15,951 beneficiary‐quarters with an unknown race/ethnicity (*n* = 1526, 303 beneficiary‐quarters)	0.58 (0.21, 0.95)
Restricting the study period to data through March 12, 2020 (*n* = 1,567,790 beneficiary‐quarters)	0.63 (0.22, 1.49)
Redefining the readmissions as a count variable (*n* = 1,510,352 beneficiary‐quarters)	0.93 (0.42, 1.44)
Including observation stays in the denominator for the readmission outcome (*n* = 1,691,037 beneficiary‐quarters)	0.69 (0.33, 1.11)

Abbreviation: CI, Confidence Interval.

## 4 Discussion

In the first three quarters following Pathways implementation, relative changes in targeted (i.e., hospital admissions) and untargeted (i.e., emergency department visits) access to care measures for CHF were similar for beneficiaries managed in and outside of the Shared Savings Program. Conversely, Pathways implementation led to a modest relative increase in 30‐day all‐cause readmissions, a care coordination measure targeted by the Shared Savings Program, in those managed by ACOs relative to controls. The relative change in the use of observation stays after discharge, an untargeted measure of care coordination, was similar in and outside of ACOs.

The finding of readmissions above is consistent with prior work, predating Pathways, noting that participation in the Shared Savings Program yielded more readmissions [5, 7]. Despite the increasing accountability implied by Pathways, failure to reduce readmissions, at least relative to those outside of the Shared Savings Program, may reflect the inability of ACOs to engage their frontline clinicians. For instance, prior research has shown that clinicians are a key component of ACOs' ability to improve quality and eliminate low‐value care [35]. Nonetheless, a quantitative study found that most clinicians (e.g., generalist physicians, internal medicine specialists, and surgeons) participating in ACOs were unaware of and unengaged with ACO objectives and activities [36]. As these clinicians continue to be reimbursed by a fee‐for‐service payment system, most do not believe that ACOs affect their compensation nor their practice patterns [36]. In this context, the risk‐bearing model may have limited sway over frontline clinician behavior and thus motivations to perform incremental work (i.e., the heavy lifting) to improve quality are muted. The Merit‐based Incentive Payment System, which mandates participation by all U.S. physicians and directly ties their Medicare payments to performance on healthcare use and quality measures, could help effect change in this regard, provided quality measures are aligned and penalties/bonuses are sufficient in magnitude [37].

Our results may also be attributed to changes in ACO enrollment (directly associated with Pathways: our data showed that 395 ACOs remained in the Shared Savings Program and 153 ACOs exited following the launch of Pathways. Of note, our exploratory analyses for the dropout ACOs showed a modest decline in quarterly observation stay rates, while rates of readmissions, hospitalizations, and emergency department visits for congestive heart failure remained unchanged following the implementation of Pathways [detailed statistical results were reported in eTable 2 in the Supplement]), particularly to the disenrollment of successful ACOs and the increasing enrollment of safety‐net providers in the Shared Savings Program. Specifically, after Pathways implementation, ACOs previously demonstrating high quality and low costs were more likely to exit the program [14]. In contrast, ACOs that included safety‐net providers, such as rural health centers, critical access hospitals, and federally qualified health centers, were more prone to join or maintain their participation in the program [38]. These ACOs may encounter significant challenges in improving quality, particularly early in their participation [39], due to their complex patient case mixes and limited clinical and non‐clinical resources available for care improvement initiatives. Moreover, prior work demonstrated that the Shared Savings Program was associated with only modest improvements in quality prior to Pathways implementation [40]. Thus, the distribution of participants (implied by dropout of high‐quality ACOs and increase in ACOs involving safety‐net providers after Pathways) may have impeded the program's ability to reduce readmissions. Policymakers should consider developing additional incentives to attract more successful ACOs to enroll continually in the Shared Savings Program and provide extra support for participating ACOs that disproportionately serve underserved populations to facilitate care improvement under the context of mandatory requirements for ACOs to assume downside risk. Otherwise, the previous distinctive benefits (e.g., improvements in quality and spending for Medicare) from the Shared Savings Program participation in a two‐sided financial risk model may be eroded.

One potential explanation for the lack of significant association between Pathways to Success and some measures of quality could be the short evaluation period. Under Pathways, ACOs transition to two‐sided risk models in less than 12 months, which may not provide sufficient time for adequate preparation for downside risk. Future research should assess the association of Pathways to Success with care quality over a more extended enrollment period to provide a clearer understanding of its long‐term effects, though influences from the pandemic will pose a significant challenge to interpretation.

Our study has several limitations. First, our findings might be biased due to unobserved confounding factors (e.g., patients' frailty and social risks). Our study used a difference‐in‐differences design with inverse probability weighting to minimize baseline differences. Further, we included group fixed effects to account for organizational‐level differences, as well as adjusted for important covariates consistent with prior work [5, 7]. Second, our analysis focuses on only 2 of the 33 quality measures targeted by the Shared Savings Program. While CHF admissions and all‐cause readmissions are among the most common measures for access to care and care coordination in Traditional Medicare [20, 41], our findings may not be generalizable to ACO performance in other domains of quality targeted by the Shared Savings Program. Nevertheless, the effects of ACOs on these outcomes have been examined previously [5, 7], allowing us to contrast the effects of Shared Savings Program overhaul (i.e., Pathways to Success implementation) with prior research. Third, this research primarily assessed the Pathways' overall impact on quality by including all ACOs in regression analyses. In reality, to attract and retain more ACOs bearing downside risk, Pathways implemented different participation policies for ACOs in the preexisting one‐ and two‐sided models [9]. Future research is needed to understand heterogeneity in effects implied by changes to ACO participation. In addition, after Pathways implementation, better‐performing ACOs withdrew from the Shared Savings Program, which might underestimate the relationship between Pathways and quality. Finally, our study was limited to the first three quarters following Pathways implementation to avoid conflation of COVID‐19 pandemic effects. Thus, our findings reflect only the initial net associations between Pathways and these measures of quality. As we evolve to a new normal in the post‐pandemic era, it will be important to measure the long‐term implications of Pathways implementation on quality.

## 5 Conclusions

Throughout the first three‐quarters of the Pathways to Success implementation, participation in the Shared Savings Program was not associated with relative changes in CHF admissions, emergency department visits for CHF, and observation stays after a hospital discharge. However, 30‐day all‐cause readmissions, a quality measure targeted by the Shared Savings Program, showed a modest increase following the launch of Pathways to Success in ACOs relative to controls. Future research should explore the long‐term association of Pathways to Success with the quality of care, accounting for pandemic perturbations.

## Conflicts of Interest

Dr. Ying reported receiving a grant from the Department of Defense during the conduct of the study. The other authors have no conflicts of interest to disclose.

## Supporting information


**Appendix S1:** Supporting Information.

## Data Availability

The data that support the findings of this study are available from The Centers for Medicare & Medicaid Services. Restrictions apply to the availability of these data, which were used under license for this study. Data are available from https://www.cms.gov/ with the permission of The Centers for Medicare & Medicaid Services.

## References

[1] Centers for Medicare and Medicaid Services , Program Data. 2023. https://www.cms.gov/medicare/medicare‐fee‐for‐service‐payment/sharedsavingsprogram/program‐data.

[2] R. Saunders , D. Muhlestein , and M. J. H. A. B. McClellan , “Medicare Accountable Care Organization Results for 2016: Seeing Improvement, Transformation Takes Time,” Health Affairs Blog (2017).

[3] M. J. Resnick , A. J. Graves , S. Thapa , et al., “Medicare Accountable Care Organization Enrollment and Appropriateness of Cancer Screening,” JAMA Internal Medicine 178, no. 5 (2018): 648–654, 10.1001/jamainternmed.2017.8087. 29554179 PMC5876897

[4] T. Borza , M. K. Oreline , T. A. Skolarus , et al., “Association of the Hospital Readmissions Reduction Program With Surgical Readmissions,” JAMA Surgery 153, no. 3 (2018): 243–250, 10.1001/jamasurg.2017.4585. 29167870 PMC5862720

[5] J. M. McWilliams , L. A. Hatfield , M. E. Chernew , B. E. Landon , and A. L. Schwartz , “Early Performance of Accountable Care Organizations in Medicare,” New England Journal of Medicine 374, no. 24 (2016): 2357–2366, 10.1056/NEJMsa1600142. 27075832 PMC4963149

[6] W. J. Mc , L. A. Hatfield , B. E. Landon , and M. E. Chernew , “Savings or Selection? Initial Spending Reductions in the Medicare Shared Savings Program and Considerations for Reform,” Milbank Quarterly 98, no. 3 (2020): 847–907, 10.1111/1468-0009.12468. 32697004 PMC7482384

[7] J. M. McWilliams , L. A. Hatfield , B. E. Landon , P. Hamed , and M. E. Chernew , “Medicare Spending After 3 Years of the Medicare Shared Savings Program,” New England Journal of Medicine 379, no. 12 (2018): 1139–1149. 30183495 10.1056/NEJMsa1803388 PMC6269647

[8] A. M. Ryan and A. A. Markovitz , “Estimated Savings From the Medicare Shared Savings Program,” JAMA Health Forum 4, no. 12 (2023): e234449, 10.1001/jamahealthforum.2023.4449. 38100095 PMC10724775

[9] Centers for Medicare and Medicaid Servcies , Final Rule Creates Pathways to Success for the Medicare Shared Savings Program. 2022. https://www.cms.gov/newsroom/fact‐sheets/final‐rule‐creates‐pathways‐success‐medicare‐shared‐savings‐program.

[10] H. H. Pham and M. B. McClellan , “ACO Quality Over Time: The MSSP Experience and Opportunities for System‐Wide Improvement,” American Journal of Accountable Care 6 (2018): e1–e15.

[11] National Association of ACOs , ACO Cost and MACRA Implementation Survey. 2023. https://naacos.memberclicks.net/assets/docs/news/naacos‐costandmacra‐survey‐5.24.2016_final.pdf.

[12] L. P. Casalino , “The Unintended Consequences of Measuring Quality on the Quality of Medical Care,” New England Journal of Medicine 341, no. 15 (1999): 1147–1150, 10.1056/nejm199910073411511. 10511617

[13] A. M. Ryan , C. M. McCullough , S. C. Shih , J. J. Wang , M. S. Ryan , and L. P. Casalino , “The Intended and Unintended Consequences of Quality Improvement Interventions for Small Practices in a Community‐Based Electronic Health Record Implementation Project,” Medical Care 52, no. 9 (2014): 826–832. 25100231 10.1097/MLR.0000000000000186

[14] M. Ying , J. H. Forman , S. Murali , et al., “Factors Affecting Accountable Care Organizations' Decisions to Remain in or Exit the Medicare Shared Savings Program Following Pathways to Success,” Health Affairs Scholar 2, no. 1 (2024): qxad093, 10.1093/haschl/qxad093. 38313161 PMC10830425

[15] Centers for Medicare and Medicaid Services , Medicare Shared Savings Program Shared Savings and Losses and Assignment Methodology Specifications. 2023. https://www.cms.gov/Medicare/Medicare‐Fee‐for‐Service‐Payment/sharedsavingsprogram/Downloads/Shared‐Savings‐Losses‐Assignment‐Spec‐V5.pdf.

[16] Dartmouth Atlas Data , Supplemental Data. 2023. https://data.dartmouthatlas.org/.

[17] Research Data Assistance Center , Medicare Shared Savings Program Accountable Care Organizations (ACO) Provider‐Level RIF. 2023. https://resdac.org/cms‐data/files/share‐saving‐program‐aco‐provider‐level.

[18] R. E. Anderson , J. Z. Ayanian , A. M. Zaslavsky , and J. M. McWilliams , “Quality of Care and Racial Disparities in Medicare Among Potential ACOs,” Journal of General Internal Medicine 29, no. 9 (2014): 1296–1304, 10.1007/s11606-014-2900-3. 24879050 PMC4139518

[19] W. B. Weeks and J. N. Weinstein , “Medicare's Per‐Beneficiary Potentially Avoidable Admission Measures Mask True Performance,” Journal of General Internal Medicine 35, no. 4 (2020): 1348–1351, 10.1007/s11606-019-05354-3. 31637656 PMC7174531

[20] Centers for Medicare and Medicaid Services , ACO #10 – Prevention Quality Indicator (PQI): Ambulatory Care Sensitive Conditions: Admissions for Heart Failure (HF). 2023. https://www.cms.gov/files/document/aco‐10‐admissions‐heart‐failure‐pdf.pdf.

[21] R. Smith‐Bindman , C. Quale , P. W. Chu , R. Rosenberg , and K. Kerlikowske , “Can Medicare Billing Claims Data Be Used to Assess Mammography Utilization Among Women Ages 65 and Older?,” Medical Care 44, no. 5 (2006): 463–470, 10.1097/01.mlr.0000207436.07513.79. 16641665

[22] Centers for Medicare and Medicaid Services , BPCI Advanced. 2024. https://www.cms.gov/priorities/innovation/innovation‐models/bpci‐advanced.

[23] Centers for Medicare & Medicaid Servcies , States Advancing All‐Payer Health Equity Approaches and Development (AHEAD) Model. 2025. https://www.cms.gov/priorities/innovation/innovation‐models/ahead. 10.1177/2752535X241259060 38825734

[24] Centers for Medicare and Medicaid Services , ACO #8 – Risk Standardized All Condition Readmission. 2023. https://www.cms.gov/medicare/medicare‐fee‐for‐service‐payment/sharedsavingsprogram/downloads/measure‐aco‐8‐readmission.pdf.

[25] M. L. F. K. Barrett , P. L. Owens , C. Stocks , C. Steiner , and M. Sheng , Identifying Observation Services in the Healthcare Cost and Utilization Project (HCUP) State Databases (Updated With 2013 HCUP Data) HCUP Methods Series Report #2016‐05 2016 https://www.cms.gov/priorities/innovation/files/fact‐sheet/bpciadvanced‐fs‐nqf1789.pdf.

[26] Z. Feng , B. Wright , and V. Mor , “Sharp Rise in Medicare Enrollees Being Held in Hospitals for Observation Raises Concerns About Causes and Consequences,” Health Affairs 31, no. 6 (2012): 1251–1259, 10.1377/hlthaff.2012.0129. 22665837 PMC3773225

[27] J. Gonzalez‐Smith , R. S. Saunders , W. K. Bleser , D. Muhlestein , and M. B. McClellan , “The Medicare Shared Savings Program in 2019: Positive Results During Major Transitions and on the Eve of A Pandemic,” Health Affairs Blog (2020), 10.1377/hblog20201016.884678. 31059355

[28] Centers for Medicare & Medicaid Servcies , Research Triangle Institute (RTI) Race Code. 2024. https://resdac.org/cms‐data/variables/research‐triangle‐institute‐rti‐race‐code.

[29] Y. Li , M. Ying , X. Cai , Y. Kim , and C. P. Thirukumaran , “Trends in Postacute Care Use and Outcomes After Hip and Knee Replacements in Dual‐Eligible Medicare and Medicaid Beneficiaries, 2013‐2016,” JAMA Network Open 3, no. 3 (2020): e200368, 10.1001/jamanetworkopen.2020.0368. 32129866 PMC7057132

[30] Chronic Condition Data Warehouse , CW Technical Guidance: Options for Determining Which CMS Medicare Beneficiaries are Dually Eligible for Medicare and Medicaid Benefits: Version 1.3. 2025. https://www2.ccwdata.org/documents/10280/19002248/ccw‐technical‐guidance‐options‐for‐determining‐dually‐eligibles.pdf.

[31] G. C. Pope , J. Kautter , R. P. Ellis , et al., “Risk Adjustment of Medicare Capitation Payments Using the CMS‐HCC Model,” Health Care Financing Review 25, no. 4 (2004): 119. 15493448 PMC4194896

[32] United States Census Bureau , American Community Survey Data. 2023. https://www.census.gov/programs‐surveys/acs/data.html.

[33] A. K. Sabbatini , K. E. Joynt‐Maddox , J. M. Liao , et al., “Accounting for the Growth of Observation Stays in the Assessment of Medicare's Hospital Readmissions Reduction Program,” JAMA Network Open 5, no. 11 (2022): e2242587, 10.1001/jamanetworkopen.2022.42587. 36394872 PMC9672971

[34] R. L. J. B. Williams , “A Note on Robust Variance Estimation for Cluster‐Correlated Data,” Biometrics 56, no. 2 (2000): 645–646. 10877330 10.1111/j.0006-341x.2000.00645.x

[35] C. L. Schur and J. P. Sutton , “Physicians in Medicare ACOs Offer Mixed Views of Model for Health Care Cost and Quality,” Health Affairs 36, no. 4 (2017): 649–654, 10.1377/hlthaff.2016.1427. 28373330

[36] A. A. Markovitz , M. D. Rozier , A. M. Ryan , et al., “Low‐Value Care and Clinician Engagement in a Large Medicare Shared Savings Program ACO: A Survey of Frontline Clinicians,” Journal of General Internal Medicine 35, no. 1 (2020): 133–141, 10.1007/s11606-019-05511-8. 31705479 PMC6957659

[37] Centers for Medicare and Medicaid Services , Learn About MIPS. 2024. https://qpp.cms.gov/mips/mvps/learn‐about‐mips.

[38] M. Ying , R. A. Hirth , P. Yan , et al., “Changes in Shared Savings Program Participation After Launch of Pathways to Success,” Journal of General Internal Medicine 38 (2022): 1780–1782, 10.1007/s11606-022-07947-x. 36441367 PMC10212830

[39] V. A. Lewis , T. Fraze , E. S. Fisher , S. M. Shortell , and C. H. Colla , “ACOs Serving High Proportions of Racial and Ethnic Minorities Lag in Quality Performance,” Health Affairs 36, no. 1 (2017): 57–66, 10.1377/hlthaff.2016.0626. 28069847 PMC5269600

[40] D. Khullar , W. L. Schpero , L. P. Casalino , et al., “Accountable Care Organization Leader Perspectives on the Medicare Shared Savings Program: A Qualitative Study,” JAMA Health Forum 5, no. 3 (2024): e240126, 10.1001/jamahealthforum.2024.0126. 38488778 PMC10943415

[41] Centers for Medicare and Medicaid Services , Hospital Readmissions Reduction Program. 2025. https://www.cms.gov/medicare/payment/prospective‐payment‐systems/acute‐inpatient‐pps/hospital‐readmissions‐reduction‐program‐hrrp.

