# Supplementary material for: Association of Pathways to Success Launch With Quality inBeneficiaries With Traditional Medicare
Source: Health Serv Res. 2025 Jul 31;61(2):e70024. doi: 10.1111/1475-6773.70024 (PMC12932017; doi:10.1111/1475-6773.70024)
Supplement: Supplementary file 1 — Appendix S1: Supporting Information. [file HESR-61-0-s001.docx]

**Supplemental Appendix Content**

**Association of Pathways to Success Launch With Quality in Beneficiaries with Traditional Medicare**

**eA.** Model Specification

**eB**. Parallel Trends Assumption Test

**eTable 1.** Results of the Parallel Trends Assumption Test for the Difference-in-Differences Design

**eFigure 1.** Unadjusted Quarterly Average Quality Outcomes for ACOs and Controls, 2018Q1-2020Q1.

**eC.** Sensitivity Analyses

**eTable 2:** Exploratory Analysis Results for Dropout ACOs

**eA.** Model Specification

Our difference-in-differences model followed the general specification, adapted from previous ACO studies:^1,2^

E($Y_{i,t,k,h})$=$\beta_{0}$+$\beta_{1}{ACO}_{k}$+$\beta_{2}{Post}_{t}$+$\beta_{3}{{ACO}_{k}\times Post}_{t}$+$\beta_{4}{ACO\_Cohort}_{k}$+$\beta_{5}{HRR}_{k}\times{Quarter}_{t}+\beta_{6}{Covariates}_{i,t}$ (1)

where Y is the quarterly quality measures (including admissions for congestive heart failure, emergency department visits for congestive heart failure, 30-day all-cause readmissions, and observation stays); “ACO” indicates if the beneficiary is attributed to an ACO; “Post” denotes the post-Pathways period; “HRR x Quarter” represents a vector of HRR by quarter fixed effects (indicators for each HRR-Quarter combination omitting a reference HRR-Quarter combination). “ACO_Cohort” represents a vector of ACO fixed effects with the control group as the reference group; “Covariates” includes all characteristics described in the Methods Section and in Table1. The quantity of interest is the estimate of “ACO x Post”, which measures the differential changes in a quality measure between the ACO group and the control group before and after Pathways implementation.

Given the voluntary nature of the Shared Savings Program, we used a propensity score weighting approach to balance the distribution of beneficiary characteristics between the ACO and control groups within each HRR, as described in a prior study.^2^ Specifically, for each study quarter, we fitted a logistic regression model predicting group status (i.e., the ACO group or the control group) as a function of beneficiary characteristics (including age, sex, race/ethnicity, Medicare and Medicaid dual eligibility, and indicators as to whether end-stage renal disease or disability were the origin of Medicare eligibility, HCC risk score) and interactions between each characteristic and HRR fixed effects. We have specified the logistic model, as follows:

${Logit(P}_{i,k})$=$\beta_{0}$+$\beta_{1}{Covariates\_Beneficiary}_{i,t}$+$\beta_{2}{Covariates\_Beneficiary}_{i,t}\times{HRR}_{k}$ (2)

From this model, we estimated a propensity score (i.e., the probability of being in the ACO group) for each beneficiary in each quarter and calculated a propensity score weight. The weight was equal to the probability of belonging to the opposite group (e.g., the probability of an ACO-assigned beneficiary being in the control group). We estimated the difference-in-differences models applying these weights.

**eB**. Parallel Trends Assumption Test

**eTable 1.** Results of the Parallel Trends Assumption Tests for the Difference-in-Differences Design, 2018Q1-2018Q3.

|  | Outcome Measures | | | |
| --- | --- | --- | --- | --- |
|  | **Admissions for CHF (targeted)** | **Emergency department visits for CHF (untargeted)** | **30-Day all cause readmissions (targeted)** | **Observation stays (untargeted)** |
|  | Coefficient (P-value) | Coefficient (P-value) | Coefficient (P-value) | Coefficient (P-value) |
| Treatment |  |  |  |  |
| ACOs | 0.014 (P=0.03) | 0.014 (P=0.06) | -0.013 (<0.001) | 0.005 (<0.001) |
| Time |  |  |  |  |
| 2018Q2 | -0.014 (P=0.005) | -0.018 (P=0.001) | 0.008 (<0.001) | 0.005 (<0.001) |
| 2018Q3 | -0.028 (P<0.001) | -0.026 (P<0.001) | 0.014 (<0.001) | 0.009 (<0.001) |
| Treatment*Time |  |  |  |  |
| ACOs*2018Q2 | **-0.005 (P=0.51)** | **-0.005 (P=0.52)** | **0.004 (0.18)** | **-0.007 (0.64)** |
| ACOs*2018Q3 | **-0.001 (P=0.93)** | **-0.007 (P=0.50)** | **0.002 (0.52)** | **-0.002 (0.28)** |

Abbreviations: Accountable Care Organizations (ACOs), Congestive Heart Failure (CHF), Quarter (Q),

The table above presents the results of the Parallel Trends Assumption tests, using patient-level generalized linear models for each outcome. All regressions included indicators for ACO participation (ACOs/controls), time (2018Q1 [referent], 2018Q2, 2018Q3), and their interaction terms. The quantities of interest are the interaction term estimates (we reported the original coefficients here), which indicate differences in pre-Pathways period trends between ACOs and controls. In addition, these models adjusted for the probability of participating in an ACO using inverse probability weight and reported coefficients for these estimates of interest. We found that the underlying assumption of the difference-in-differences design was not violated, as the bolded interaction term coefficients were statistically insignificant under a 2-tailed test with P<0.05.

**eFigure 1.** Unadjusted Quarterly Average Quality Outcomes for ACOs and Controls, 2018Q1-2020Q1.

Notes: The dotted vertical line represents the effective date of Pathways to Success on July 1^st^, 2019.

The Figure above illustrates that between the first quarter of 2018 and the third quarter of 2018, the unadjusted average quarterly quality outcomes for the ACOs and controls followed similar trends, further confirming the validity of the difference-in-differences design.

**eC.** Sensitivity Analyses

The results of the sensitivity analyses are summarized in Table 3. To evaluate the robustness of our main findings, we conducted a series of sensitivity tests. Specifically, we examined whether the primary results were robust when using alternative definitions of the pre-Pathways period, as the difference-in-differences model was sensitive to how the preintervention periods were defined. We therefore re-estimated models for each quality outcome using alternative preintervention periods: July 1, 2017, to March 31, 2018, and July 1, 2017, to September 30, 2018. We also re-estimated the outcomes by extending the post-Pathways period to begin on January 1, 2019 and end on March 31, 2020, to assess whether there was any preemptive response following the official announcement of Pathways to Success on December 21, 2018.

In addition, we tested the robustness of our findings to alternative model specifications by analyzing all outcomes with generalized linear models using a Gaussian family function and an identity link. We included individuals with unknown race/ethnicity (n = 15,951 beneficiary-quarters) to determine whether our results were sensitive to changes in the sample size. Given that the White House officially declared COVID-19 a global pandemic on March 13, 2020, we limited our study period to data through March 12, 2020 to reduce potential bias related to the pandemic. We further assessed the robustness of our findings by modifying the outcome definitions. Specifically, by converting binary outcomes to count outcomes and by including observation stays in the denominator for the readmission outcome, following prior research.^3^

Across all these tests, the results were consistent with those from the difference-in-differences models, confirming the robustness of our findings.

eTable 2: Exploratory Analysis Results for Dropout ACOs

|  | Adjusted differential change  (95% CI) | P-value |
| --- | --- | --- |
| Admissions for CHF (targeted) | -1.02  (-9.15, 7.11) | 0.81 |
| Emergency department visits for CHF (untargeted) | -1.12  (-11.00, 8.76) | 0.82 |
| 30-Day all cause readmissions (targeted) | -2.09  (-9.92, 5.73) | 0.60 |
| Observation stays (untargeted) | -0.67  (-1.13, -0.21) | 0.004 |

**References:**

1. McWilliams JM, Hatfield LA, Landon BE, Hamed P, Chernew ME. Medicare Spending after 3 Years of the Medicare Shared Savings Program. *New England Journal of Medicine*. 2018/09/20 2018;379(12):1139-1149. doi:10.1056/NEJMsa1803388

2. McWilliams JM, Hatfield LA, Chernew ME, Landon BE, Schwartz AL. Early Performance of Accountable Care Organizations in Medicare. *New England Journal of Medicine*. 2016/06/16 2016;374(24):2357-2366. doi:10.1056/NEJMsa1600142

3. Sabbatini AK, Joynt-Maddox KE, Liao JM, et al. Accounting for the Growth of Observation Stays in the Assessment of Medicare's Hospital Readmissions Reduction Program. *JAMA network open*. Nov 1 2022;5(11):e2242587. doi:10.1001/jamanetworkopen.2022.42587
